# Inflammatory rhabdomyoblastic tumor, pheochromocytoma, and pulmonary hamartoma in a patient with neurofibromatosis type 1: a case report

**DOI:** 10.1186/s13000-024-01503-3

**Published:** 2024-06-11

**Authors:** Otto Jokelainen, Heidi Myllykangas, Katri Rajala, Jarkko Marttila, Reijo Sironen

**Affiliations:** 1 Institute of Clinical Medicine, Pathology and Forensic Medicine, University of Eastern Finland, Kuopio, Finland; 2 Department of Clinical Pathology, Diagnostic Imaging Center, Kuopio University Hospital, Kuopio, Finland; 3 Department of Plastic Surgery, Kuopio University Hospital, Kuopio, Finland; 4 Department of Clinical Genetics, Kuopio University Hospital, Kuopio, Finland; 5 Department of Clinical Radiology, Kuopio University Hospital, Kuopio, Finland

**Keywords:** Neurofibromatosis type 1, Inflammatory rhabdomyoblastic tumor, Pheochromocytoma, Pulmonary hamartoma

## Abstract

**Background:**

Inflammatory rhabdomyoblastic tumors are relatively recently recognized soft tissue tumors with a low malignant potential. Here, we present a case of concurrent inflammatory rhabdomyoblastic tumor (IRMT), adrenal pheochromocytoma, and pulmonary hamartoma in a patient with neurofibromatosis type 1 (NF1). To our knowledge, this is the first time that this constellation of tumors has been described in the literature.

**Case presentation:**

A female patient in her late 20s with known NF1 was diagnosed with an inflammatory rhabdomyoblastic tumor, pheochromocytoma, and pulmonary hamartoma in a short succession. IRMT was found to harbor a near-haploid genome and displayed a typical immunohistochemical profile as well as a focal aberrant p53 expression pattern.

**Conclusions:**

This case report strengthens the theory that defects in the tumor suppressor NF1 play a central role in the pathogenesis of inflammatory rhabdomyoblastic tumors and that IRMT may be part of the spectrum of neurofibromatosis type 1 related tumors.

## Introduction

Neurofibromatosis type 1 (NF1) is a neurocutaneous disorder that clinically manifests as cutaneous neurofibromas, café-au-lait macules, skinfold freckling, and ocular Lisch nodules. In addition, other central and peripheral nerve tumors such as optic gliomas and plexiform neurofibromas are common. This syndrome is one of the most common autosomal dominant disorders, with a reported incidence of 1 in 3000 births. It is estimated that 30–50% of cases occur sporadically with the appearance of *de novo* mutations. The syndrome is caused by loss-of-function variants of the tumor suppressor NF1 gene located on chromosome 17q11.2. This gene encodes neurofibromin, a negative regulator of the Ras proto-oncogene. The loss of neurofibromin leads to increased RAS activity and excessive cell division. Neurofibromin is primarly expressed in neurons, Schwann cells, oligodendrocytes, and astrocytes [1, 2].

Inflammatory rhabdomyoblastic tumor (IRMT) is a proposed unifying term used to encompass overlapping cases that were initially described by Merchant et al. as inflammatory leiomyosarcomas, and those cases coined as “histiocyte-rich rhabdomyoblastic tumors” (HRRMT) by Martinez et al. [3–5]. This relatively novel group of tumors usually presents as soft tissue masses in the lower extremities and trunk of young and middle-aged patients with male predominance. It is characterized by a near-haploid karyotype, distinct histology, and evidence of muscle differentiation on immunohistochemistry. Most patients follow an indolent course. A small proportion of patients were previously diagnosed with neurofibromatosis type 1 [5, 6]. A recent sequencing study by Odate et al. showed that most tumors (5/8) harbor pathogenic mutations in NF1 [7].

Pheochromocytoma, also known as intra-adrenal paraganglioma, is a rare neuroendocrine neoplasm originating from the chromaffin cells of the adrenal medulla. The tumor is characterized by the secretion of excess catecholamines, manifesting as headaches, palpitations, hypertension, and excess diaphoresis. These tumors confer a non-negligible risk of metastasis (approximately 5–15%); therefore, all pheochromocytomas are considered malignant [8]. Pheochromocytoma occurs in approximately 1–5% of patients with neurofibromatosis type 1, and these patients have a tenfold higher incidence of pheochromocytomas than the general population [9]. The mutational profile of NF1-associated pheochromocytomas appears to differ from that of sporadic pheochromocytomas [10].

Pulmonary hamartomas, in contrast to their names, are true neoplasms. They are usually solitary, well-circumscribed nodules composed of at least two mesenchymal elements and trapped respiratory epithelium. Most tumors were peripheral, with a minority (10%) located centrally in the bronchi. Its clinical course is usually benign, and recurrence and malignant transformation are rare [11]. Neurofibromatosis type 1 confers a higher risk of developing cystic lung disease; however, no case series or population-based studies concerning pulmonary hamartomas and NF1 exist [1]. A single case of intrabronchial pulmonary hamartoma in an NF1 setting has been previously reported [12].

## Case presentation

A female in her late 20s was initially diagnosed with neurofibromatosis type 1 in childhood. There was a known inheritance pattern in the patient’s family, and she carried a pathogenic germline heterozygous NF1-mutation at c.6494 C > G, p.(Ser2165*). Clinically, she presented with café au-lait spots, fold lentiges, and multiple subcutaneous tumors. Besides the classical presentation of neurofibromatosis and menstrual pain attributed to the two uterine leiomyomas, the patient was previously healthy. She had been clinically followed up for NF1 up to the age of 18 years, but these follow-ups had ceased.

### Clinical history

The patient was referred to our hospital for multiple painful subcutaneous tumors, the largest of which was in the inner thigh, measuring up to 4 cm. Surgery was scheduled for the removal of the lesions. During the surgery, while under general anesthesia, she experienced a high systolic blood pressure of up to 200 mmHg and wide-complex tachycardia of up to 150 beats per minute. Skin erythema was also observed. Anesthesia was intensified and the symptoms subsided. Surgery was completed as planned, and 12 tumors were subjected to pathological examination. These were diagnosed as benign conventional neurofibromas according to World Health Organization (WHO) criteria [13].

**Fig. 1:**
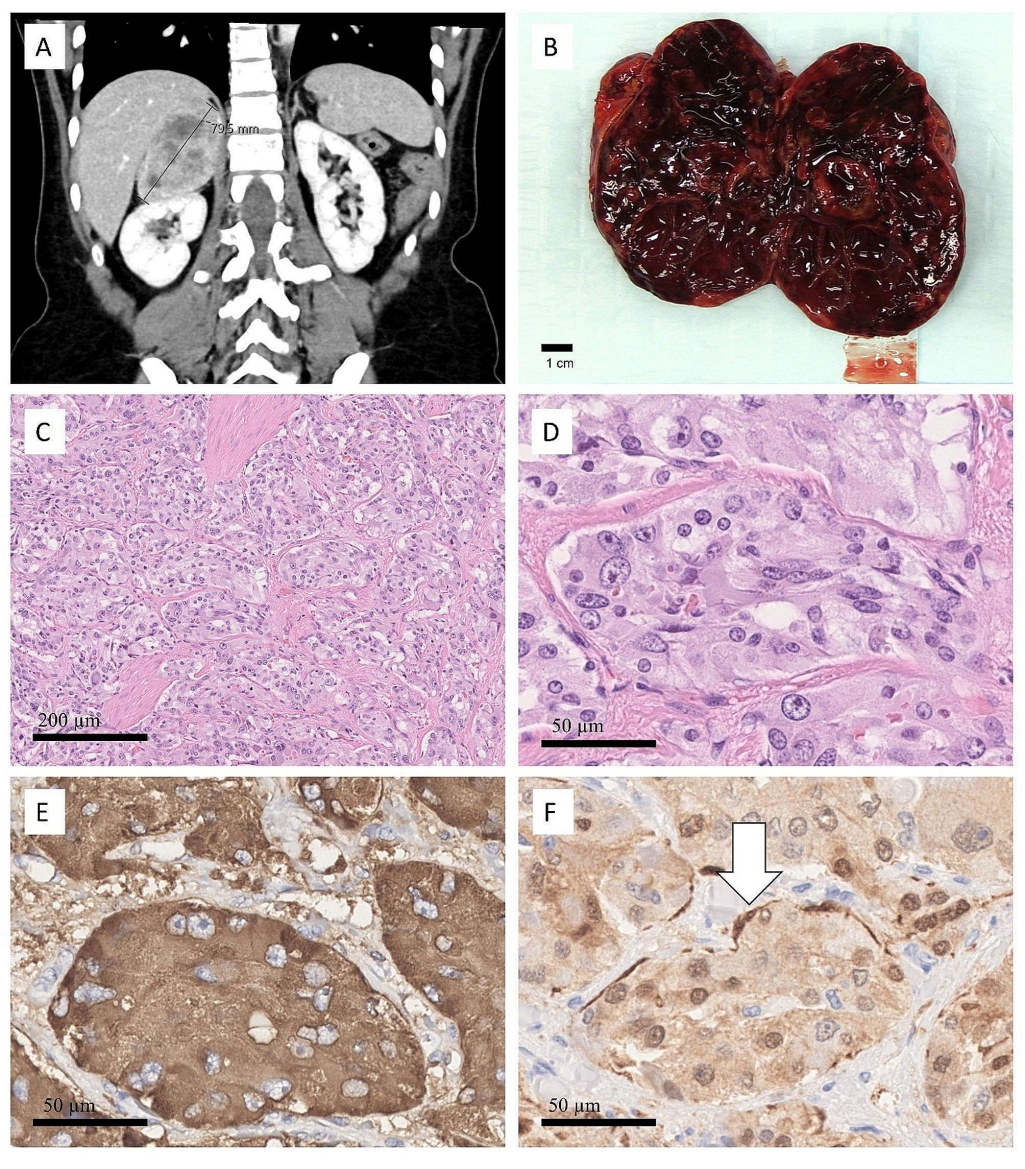
Radiological, macroscopic, and histological features of pheochromocytoma. Contrast-enhanced computed tomography showing mild enhancement in the tumor, replacing the right adrenal gland **(A)**. Macroscopically, the tumor is well-defined, soft, and hemorrhagic **(B)**. The tumor had a nested “Zellballen” growth pattern, with fibrous septa and hemorrhage dividing the nests **(C)**. Tumor cells were variable in size and polygonal with abundant pinkish cytoplasm. There were single prominent nucleoli in larger cells **(D)**. Tumor cells are strongly and diffusely positive for synaptophysin (picture) and chromogranin (not shown) **(E)**. Occasional S100 positive sustentacular cells (arrow) can be identified at the periphery of nests **(F)**

After the surgery, she underwent cardiac MRI, where no structural abnormalities were found. However, there was a large heterogeneous mass, which in the subsequent abdominal MRI was confirmed to be a left adrenal mass measuring 7.4 × 6.3 × 4.4 centimeters. Hormonal testing revealed elevated metanephrine (1.2 nmol/L) and normetanephrine (18.0 nmol/L) levels, raising suspicion of pheochromocytoma. The patient was started on phenoxybenzamine and surgery was scheduled. Preoperative abdominal computed tomography revealed a 6 × 3 × 2.7 cm cystic and solid tumor within the right gluteus minimus muscle. Retrospectively, according to abdominal CT, this tumor had existed already 7 months before, with no growth in this time. Open surgery for the adrenal tumor was commenced, and the left adrenal gland containing the tumor was removed. The tumor was interpreted by a pathologist as a pheochromocytoma according to the WHO criteria (Fig. 1) [8].

Otherwise uneventful post-surgery recovery was hindered by the Covid-19-infection, during which thoracic radiography was performed. This revealed a 1,4 cm diameter left upper lobe tumor, which was confirmed by pulmonary CT. These findings were followed by ^68^Ga-DOTANOC PET-CT, which showed above-background uptake in both lung (SUVmax 6,2) and gluteal (SUVmax 5,0) tumors. The gluteal tumor was radiologically suspected to be a neurofibroma or malignant peripheral nerve sheath tumor, and core needle biopsy was performed. Core needle biopsy was difficult to interpret, but the pathologist suspected a high-grade sarcoma. The final diagnosis was not obtained from the biopsy. Biopsy findings prompted clinicians to suspect lung metastasis. Subsequently, the gluteal tumor was surgically resected, and a right upper lung lobectomy was performed separately. Postoperative recovery was uneventful, and the patient underwent hysterectomy for histopathologically confirmed leiomyomas. The patient is disease-free after 15 months after diagnosis.

### Histopathology

#### Pheochromocytoma

The cut surface of the adrenal tumor showed a well-circumscribed hemorrhagic tumor. The adrenal tumor is composed of plump epithelioid cells with an abundant eosinophilic cytoplasm and nested “Zellballen” growth pattern. The nuclei showed moderate variation in size, clumped chromatin, and small conspicuous nucleoli. Marked intratumoral hemorrhage was observed. The tumor cells stained positive for chromogranin and synaptophysin and weakly and heterogeneously stained with S100. In addition, occasional S100 positive sustentacular cells were identified, rimming the tumor cell nests (Fig. 1).

#### Inflammatory rhabdoid tumor

**Fig. 2:**
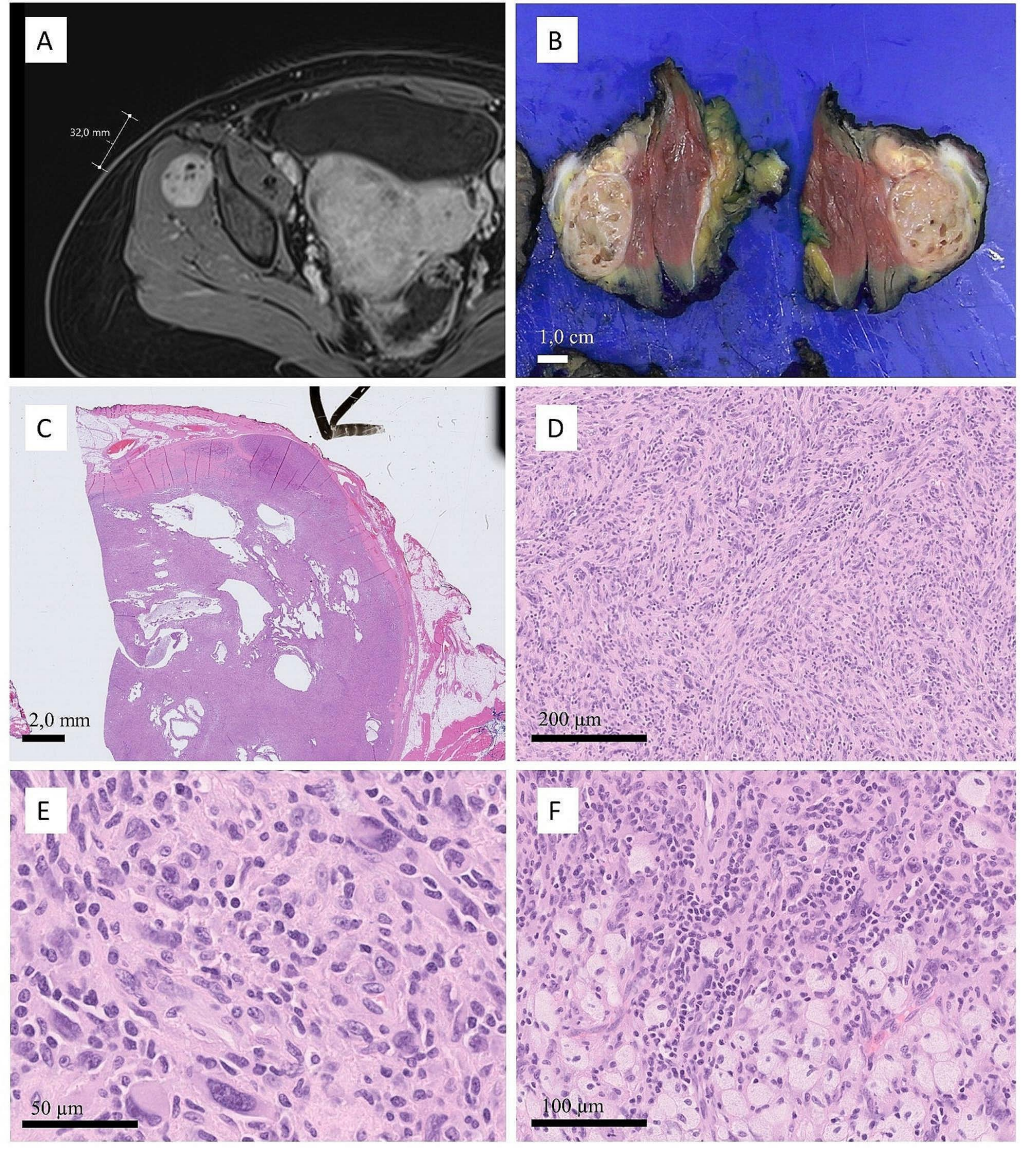
Radiological, macroscopic, and histological features of inflammatory rhabdomyoblastic tumor. Post-contrast T1 fat saturation MRI showing a well-circumscribed, strongly enhanced solid and cystic tumor within the gluteus minimus muscle **(A)**. Macroscopically, the tumor was beige in color with small cystic spaces. The surgical margin at the bottom of the resection is slim **(B)**. The low-power field of the tumor section showed a fibrous pseudocapsule. The tumor bulges through the capsule at the top-right corner **(C)**. Tumor cells growing in short intersecting fascicles **(D)**. Tumor cells showed large hyperchromatic nuclei with occasional multinucleation, prominent nucleoli, and abundant eosinophilic cytoplasm. There are many histiocytes and lymphocytes intermixed with larger tumor cells **(E)**. Focal areas of foamy histiocytes are identified **(F)**

The intragluteal tumor was well circumscribed and had a fibrous pseudocapsule. The cut surface revealed a gray-tan tumor with small cystic spaces. Histological examination revealed a predominantly solid tumor with frequent cystic growth. The tumor was composed of partly spindly hyperchromatic mononuclear cells with abundant eosinophilic cytoplasms. Large pleomorphic and multinucleated cells rimmed cystic spaces. Variable numbers of lymphocytes and histiocytic cells were observed intermixed with the tumor cells, and focal aggregates of foam cell histiocytes were also observed at the edge of the tumor. No tumor necrosis was observed. The tumor was completely resected with < 0,1 cm marginal resection margins without the accompanying fascia (Fig. 2).

**Fig. 3:**
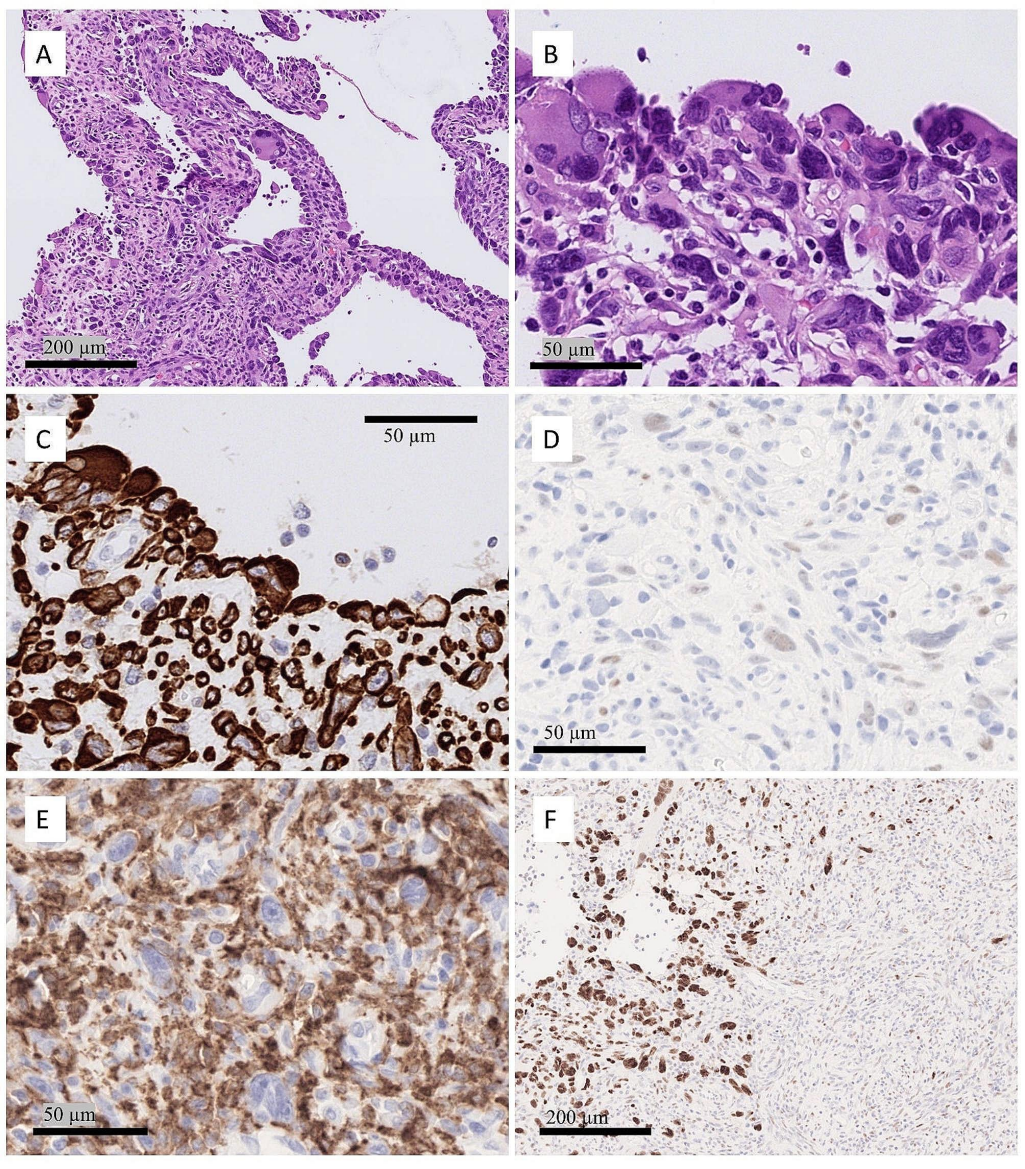
Histological and immunohistochemical features of an inflammatory rhabdomyoblastic tumor. Large cystic spaces within the tumor, showing large pleomorphic cells rimming the cystic spaces **(A)**. Large multinucleated rhabdoid cells rimming the cystic spaces **(B)**. The tumor cells were strongly and diffusely positive for desmin **(C)**. Core needle biopsy showed scattered MyoD1 positive tumor cells **(D)**. Many intermixed histiocytic cells were identified by CD163 **(E)**. P53 immunostain showing patchy aggregates of strongly and diffusely positive tumor cell clustering around cystic spaces. Solid areas show a heterogeneous wild-type staining pattern **(F)**

Tumor cells were strongly and diffusely positive for desmin and patchy positivity for SMA was seen. The core needle biopsy sample showed weak MyoD1 positivity in scattered cells; however, the surgical sample was completely negative for MyoD1 despite repeated staining from another block. This discrepancy may be due to inadequate fixation. The large multinucleated cells palisading around the cystic spaces were strongly and diffusely positive for p53, whereas the cells in the solid areas showed a wild-type expression pattern. CD68 and CD163 showed an abundant population of histiocytic cells intermixed with tumor cells (Fig. 3). Ki-67 was low at 5%. Mitotic figures were inconspicuous in 2/50 high-power fields (11,8 mm^2^) at 40x magnification. All other markers, including S100, pancytokeratin, caldesmon, pancytokeratin AE1/AE3, ALK-1, SMMHC, calponin, MUC4, GFAP, SOX10, Melan A, and HMB45, were negative. H3K27me3 showed normal nuclear expression. Thus, an inflammatory rhabdoid tumor was diagnosed according to WHO criteria [14].

The IRMT was subjected to further diagnostic analyses. RNA was extracted from paraffin slides, and a sequencing library was constructed using the Archer FusionPlex Sarcoma v2 and Archer FusionPlex CTL panels. The panels identified both novel and unknown fusions with 63 and 17 predetermined genes, respectively. The final libraries were sequenced using Ion Torrent semiconductor sequencing. The Archer Analysis software was used to interpret the results. No gene fusions were detected.

**Fig. 4:**
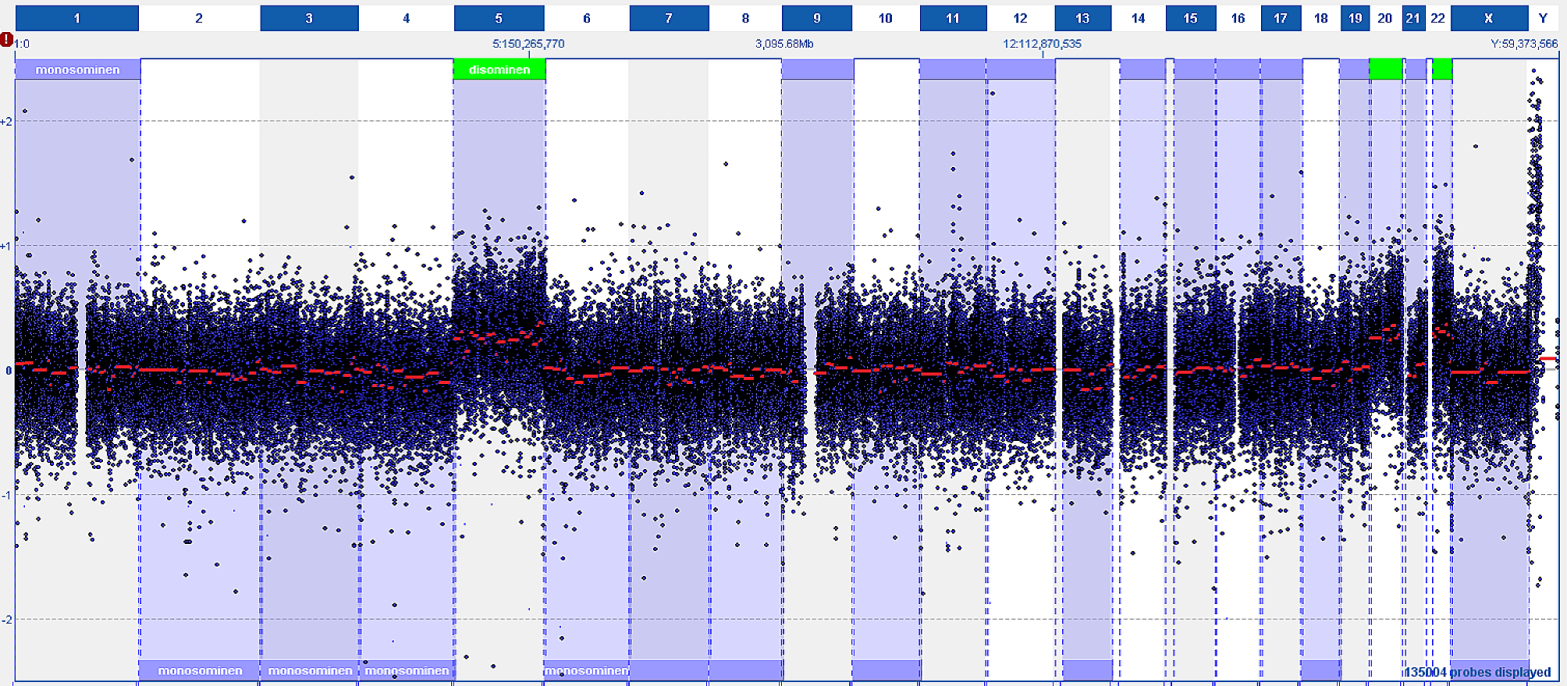
Chromosomal microarray profile of inflammatory rhabdomyoblastic tumor. The copy number was displayed on a weighted log2 ratio plot. Chromosomes 1–22, X, and Y are shown from left to right, respectively. The whole-genome view shows a relatively high copy number ratio for chromosomes 5, 20, and 22, consistent with widespread loss of heterozygosity and near-haplodization

Comparative genomic hybridization was performed using the OGT Consortium Cancer + SNP 180k microarray. The relative copy number changes were read using a laser scanner and analyzed using Cytosure Interpret-software (Hg 19). This result showed 30–40% of the cells had a hyperhaploid karyotype, with chromosomes 5, 20, and 22 having a relatively higher copy number corresponding to a normal disomic karyotype (Fig. 4).

#### Pulmonary hamartoma

**Fig. 5:**
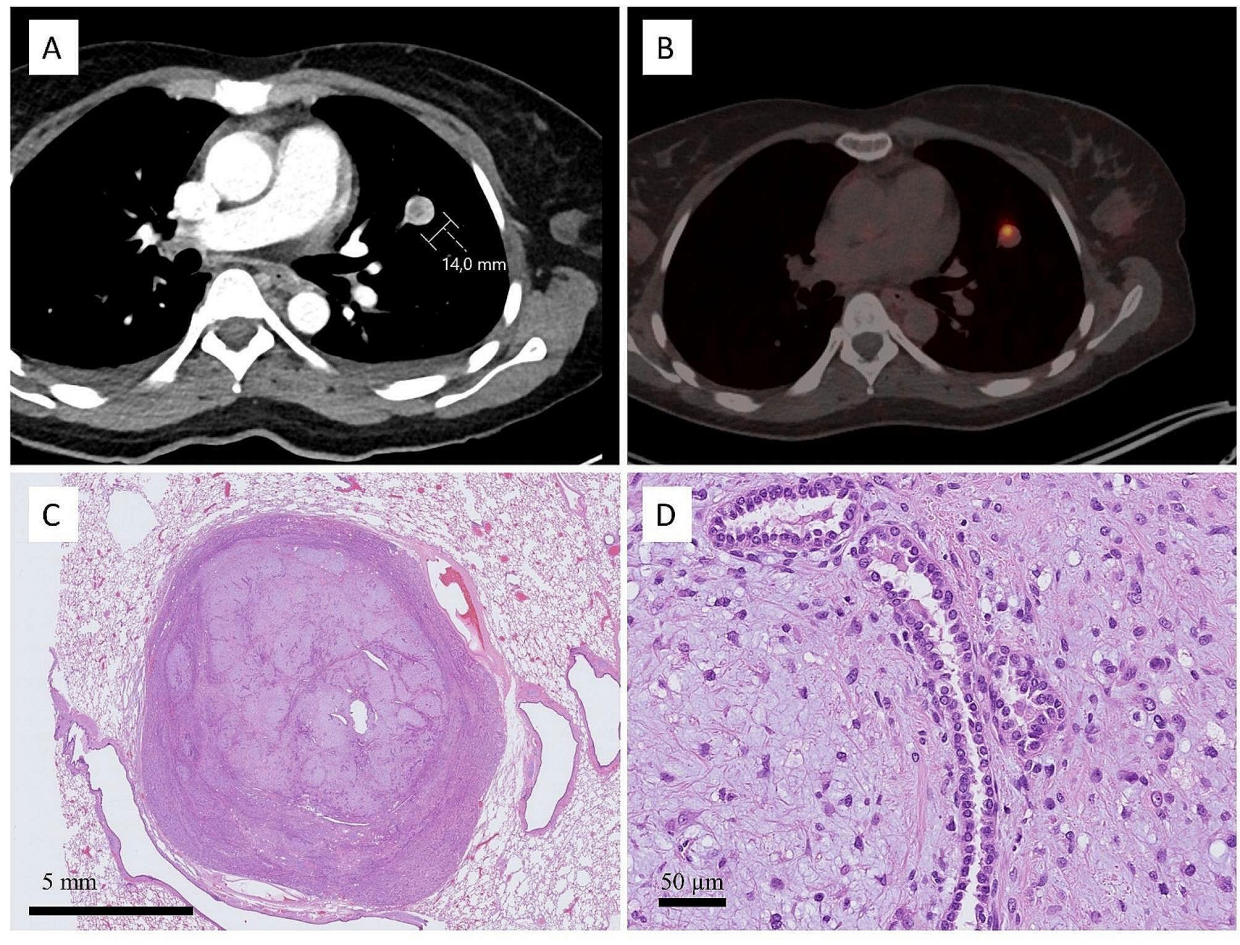
Radiological and histological features of pulmonary hamartoma. Contrast-enhanced computed tomography revealed a well-circumscribed 14 mm diameter tumor within the upper light lobe. There was mild heterogenous contrast enrichment **(A)**. ^68^Ga-DOTANOC PET-CT showing above-background uptake of radionuclide with SUVmax of 6,2 **(B)**. Tumor sections show well-circumscribed tumor with nodular structures in the middle and condensed glandular structures at the periphery **(C)**. Nodules were composed of stellate to epithelioid cells with at most mild atypia in myxoid background. The entrapped bronchiolar epithelium is readily visible **(D)**

The lung tumor was well-circumscribed and histologically composed of nodular aggregates of epithelioid to stellated cells within the basophilic myxoid stroma. Entrapped non-atypical respiratory epithelial cells were frequently observed between the nodules. Occasional intermixed mature adipocytes were also observed. No mature terminally differentiated chondroid component was observed in this case. Immunohistochemical findings showed that the stellate cells were strongly and diffusely S100-positive, supporting their chondroid background. Pancytokeratin AE1/AE3, CD34, and EMA were negative. Occasional scattered cells showing smooth muscle differentiation (SMA + and desmin+) were observed among the stellate cells. Ki67 proliferation was very low < 1–2%. The entrapped epithelial strands showed strong positivity for TTF1 and scattered p63-positivity. Pulmonary hamartoma was diagnosed according to the WHO criteria (Fig. 5) [11].

## Discussion and conclusions

Our case illustrates a constellation of rare tumor entities in a single patient with NF1, in addition to conventional syndrome-related neurofibromas. Although there is a known disposition to develop pheochromocytomas in NF1, the incidence of IRMT and pulmonary hamartomas remains obscure. Considering the relatively high percentage of NF1 patients in previously published case series of IRMT, and the frequency of NF1 mutations found, it is likely that the NF1 gene plays an important role in the pathogenesis of these tumors (Table 1) [4–7, 15, 16]. Germline or sporadic mutations in NF1, combined with the loss of heterozygosity (LOH) that occurs during haploidization events, could be the driving factors for tumorigenesis.

**Table 1:** Literature review of published cases of inflammatory rhabdomyoblastic tumors, NF1 mutations, p53 mutations and neurofibromatosis type 1

Publication	NF1 mutated tumors (%)	TP53 mutated tumors (%)	Tumors in NF1 context (%)	Number of cases
Odate T et al. (2023)	6 (46.2%)	4 (30.1%)	2 (15.4%)	13
Sukhanova M et al. (2022)	2 (100%)	2 (100%)	0	2
Coultier J et al. (2021)	NA	NA	1 (13.3%)	6*
Lee J et al. (2021)	NA	NA	1 (25%)	4
Martinez A et al. (2019)	2 (20%)	0	0	10
Arbajian E et al. (2018)	2 (25%)	1 (12.5%)	0	8

*Excluding cases published by Martinez and Arbajian

NA, not available

Curiously, concurrent TP53-mutations with lower allele frequencies were identified in many NF1-mutated cases, suggesting the emergence of a subclonal population within the tumors [7]. This was supported by our finding that large, atypical cells rimming the cystic spaces were strongly and diffusely positive for p53, indicating a mutated phenotype. A case of high-grade transformation to rhabdomyosarcoma has been reported, and the rhabdomyosarcomatous component harbored the LOH of TP53, indicating that cases with TP53 loss might have a more aggressive course [17]. However, this remains to be confirmed.

This study supports the theory that NF1 plays an important role in IRMT tumorigenesis and that IRMT may be a part of NF1 related tumors. Further studies are required to elucidate the molecular mechanisms underlying the concurrent loss of NF1 function and TP53 mutations. This could pave the way for better diagnosis, prognostication, and treatment of these patients.

## Data Availability

No datasets were generated or analysed during the current study.
